# Prognostic value of phosphorylated Raf kinase inhibitory protein at serine 153 and its predictive effect on the clinical response to radiotherapy in nasopharyngeal carcinoma

**DOI:** 10.1186/s13014-016-0696-5

**Published:** 2016-09-20

**Authors:** Siwei Li, Taowen Liu, Wenfa Mo, Qiaoyan Hou, Yingqiong Zhou, Meilian Liu, Zhoukai He, Zhengchun Liu, Qiuqiu Chen, Hua Wang, Xiang Guo, Weixiong Xia, Musheng Zeng, Haiyun Zhao

**Affiliations:** 1 Department of Radiation Oncology, The Affiliated Hospital of Guilin Medical University, No. 15 Lequn Road, Guilin, 541004 Guangxi Zhuang Autonomous Region People’s Republic of China; 2 Department of Oncology, Nanxishan Hospital of Guangxi Zhuang Autonomous Region, No.46 Chongxin Road, Guilin, 541004 Guangxi Zhuang Autonomous Region People’s Republic of China; 3 Department of Pathology, The Affiliated Hospital of Guilin Medical University, No. 15 Lequn Road, Guilin, 541004 Guangxi Zhuang Autonomous Region People’s Republic of China; 4 Department of Otorhinolaryngology, Head and Neck Surgery, The Affiliated Hospital of Guilin Medical University, No. 15 Lequn Road, Guilin, 541004 Guangxi Zhuang Autonomous Region People’s Republic of China; 5 State Key Laboratory of Oncology in Southern China, Collaborative Innovation Center for Cancer Medicine, Sun Yat-sen University Cancer Center, No.651 Dongfeng Road East, Guangzhou, 510060 People’s Republic of China; 6 Department of Nasopharyngeal Carcinoma, State Key Laboratory of Oncology in Southern China, No.651 Dongfeng Road East, Guangzhou, 510060 People’s Republic of China; 7 Department of Otorhinolaryngology, Head and Neck Surgery, Nanxishan Hospital of Guangxi Zhuang Autonomous Region, No.46 Chongxin Road, Guilin, 541004 Guangxi Zhuang Autonomous Region People’s Republic of China

**Keywords:** Phosphorylated Raf kinase inhibitor protein (pRKIP), Nasopharyngeal carcinoma, Radiotherapy, Sensitivity, Prediction, Prognosis

## Abstract

**Background:**

Radiation is an effective treatment against nasopharyngeal carcinoma (NPC). However, radioresistance-induced locoregional recurrence remains as a major cause of treatment failure. Therefore, radiosensitivity indicators prior to treatment should be developed to screen radioresistant patients. Previous studies revealed that RKIP (Raf kinase inhibitor protein) is associated with NPC prognosis and radiosensitivity. However, the relationship of p-Ser153 RKIP (RKIP in a phosphorylated form at residue serine153) expression with the effect of radiation and prognosis of NPC patients is not elucidated. Thus, these clinical implication of the phosphorylated RKIP in NPC has yet to be described.

**Methods:**

The effect of p-Ser153 RKIP on locoregional relapse-free survival (LRRFS) was first analyzed in a retrospective cohort of NPC patients without distant metastasis at initial diagnosis. They received radical intensity-modulated radiotherapy alone. Of 180 patients were enrolled in the ongoing matched pair study. The patients were re-classified into radioresistant group or radiosensitive group on the basis of the specified criteria. Patients in the two groups were matched in terms of radiosensitivity-related factors. p-Ser153 RKIP was examined by immunohistochemical staining on a NPC tissue microarray before radiotherapy. The relationship between the expression of p-Ser153 RKIP and the effect of radiotherapy was also analyzed.

**Results:**

In this study, a retrospective cohort with 733 cases who received radical radiotherapy alone was established. Using the cohort, we validated that the p-Ser153 RKIP expression observed through immunohistochemical staining in a pretreatment NPC tissue microarray was an independent prognostic factor of LRRFS and OS; we also confirmed that endemic patients with a positive p-Ser153 RKIP expression benefited from irradiation alone in terms of locoregional relapse-free survival. A total of 180 patients were enrolled in a matched pair study. Both groups were well matched in terms of radiosensitivity-related factors. On the basis of the p-Ser153 RKIP expression, we predicted the following data: 80.0 % sensitivity, 73.3 % specificity, 76.7 % accuracy, 75.0 % positive predictive value, and 78.6 % negative predictive value.

**Conclusions:**

Our results revealed for the first time that positive p-Ser153 RKIP expression was a favorable prognostic factor. It was also positively correlated with the radiosensitivity of NPC. p-Ser153 RKIP could also be used as a biomolecular marker with good availability and authenticity to preliminarily screen NPC-related clinical radiosensitivity.

**Electronic supplementary material:**

The online version of this article (doi:10.1186/s13014-016-0696-5) contains supplementary material, which is available to authorized users.

## Introduction

Radical radiation is an effective treatment against NPC; however, locoregional recurrence remains as a major cause of the failure of treatments for locoregionally advanced NPC [1]. As such, local control should be enhanced to improve survival and prognosis [1]. Therefore, radiosensitivity indicators prior to treatment should be developed on the basis of simple, reliable, and effective methods to screen radioresistant patients. These indicators provide a reasonable basis of specific NPC radiotherapy.

RKIP, a well-established metastasis suppressor gene, has been identified as a negative regulator of survival signals induced by the activation of MAPK and NF-kB pathways. The overexpression of RKIP reverses tumor cell resistance to apoptosis by various factors, such as irradiation [2, 3], chemotherapeutic drugs [4, 5], and TRAIL [6]. RKIP is also considered as a prognostic marker of several non-head–neck cancers [7–11]. Furthermore, the RKIP protein is associated with the metastasis, progression, and prognosis of NPC [12].

The functional activities of RKIP are regulated by its phosphorylation status on serine 153 [13]. In contrast to the unphosphorylated form, p-Ser153 RKIP (RKIP in a phosphorylated form at residue S153) antagonizes the actions of the unphosphorylated RKIP, which inhibits survival signals and promotes apoptosis. It is because p-Ser153 RKIP fails to effectively bind to Raf-1, p-Ser153 RKIP does not inhibit the MAPK signaling pathway [14].

Limited data are available to explore the clinical implication of p-Ser153 RKIP in tumors. For example, the loss or reduction of p-Ser153 RKIP expression in breast cancer [15] is associated with poor disease-free survival; furthermore, the complete loss of p-Ser153 RKIP is an independent prognostic factor. In patients with early-stage lung cancer or in elderly individuals, the normal expression of phospho-RKIP is a predictive indicator of a more favorable survival than the reduced expression of phospho-RKIP [16]. In contrast to these tumors, other tumors, such as multiple myeloma (MM) and stage II colon cancer, exhibit p-Ser153 RKIP that may positively contribute to the overall cell survival and drug resistance of MM through the constitutive activation of survival pathways and the downstream transcription of anti-apoptotic genes [17, 18]. Considering the varied results in different tumors, we should investigate the clinical implication of the phosphorylated RKIP in NPC. However, studies have yet to describe the relationship of p-Ser153 RKIP expression with the prognosis of NPC patients and the effect of radiation on NPC.

In this study, the effect of pre-RT p-Ser153 RKIP expression in a NPC tissue microarray on locoregional relapse-free survival (LRRFS) was examined. The study was based on patients from an established retrospective cohort composed of 733 cases who received irradiation alone. In the ongoing matched pair study, the patients were re-classified into radioresistant group or radiosensitive group on the basis of the specified criteria. This study also determined whether p-Ser153 RKIP is closely related to NPC radiosensitivity to investigate the predictive significance of p-Ser153 RKIP as a biomarker in the preliminary screening of the clinical response to radiotherapy of NPC.

## Materials and methods

### Patients and general data

A total of 733 patients with biopsy-proven NPC from January 2005 to December 2006 were enrolled in this study. The patients did not manifest distant metastasis at initial diagnosis. Patients with sufficient pretreatment tumor biopsy specimens were initially subjected to radical radiotherapy alone through intensity-modulated radiotherapy (IMRT) at the Sun Yat-sen University Cancer Center. Patient consent and approval from the Institute Research Ethics Committee were acquired before these clinical materials were used for research. Clinical follow-up data were obtained from the patients’ medical records.

A patient was considered eligible for this study if the following criteria were satisfied before treatment was administered: (1) histologically confirmed non-keratinizing or undifferentiated NPC (World Health Organization [WHO] type II or type III cancer); (2) Stage I to Stage IV_(A–B)_ according to the International Union Against Cancer (UICC) TNM classification (2002); (3) performance status ranging from 0 to 2 in accordance with the Eastern Cooperative Oncology Group system; (4) absence of serious renal dysfunction, as demonstrated by serum creatinine of <1.5 mg/dL and a creatinine clearance rate of at least 60 mL/min; (5) absence of serious liver dysfunction, as demonstrated by bilirubin level of <1.5 mg/dL, with a ratio of aspartate aminotransferase to alanine aminotransferase of < twofold the normal upper limit; and (6) adequate hematologic function, as demonstrated by a hemoglobin concentration of ≥100 g/L, a leukocyte count of ≥4.0 × 10^9^/L, and a platelet count of ≥100 × 10^9^/L.

Patients were excluded from this study on the basis of the following criteria: (1) pathology types that were not WHO II/III NPC; (2) distant metastasis under clinical examination, imaging, or biopsy proven; (3) with any form of chemotherapy; (4) previous radiation therapy to the head and/or neck region; (5) previous surgery on the primary tumor site or neck with the exception of diagnostic biopsy; (6) history of malignant tumors or multiple tumors occurring at the same time; and (7) disease occurring during pregnancy.

### Reclassification of patients into the radioresistant group or the radiosensitive group according to the following criteria

The enrolled patients did not receive treatment against disease prior to biopsy, with a minimum follow-up of 5 years following the beginning of radiotherapy. The patients were classified into two groups according to the following criteria. Patients with biopsy-proven recurrent NPC occurring at the original anatomical site of the nasopharynx and/or the neck within 5 years of a radiotherapy course were classified as the radioresistant group. The pathological type at relapse was the same as the previous type before treatment. Patients with a minimum follow-up of 5 years following the beginning of radiotherapy, without evidence of recurrence both at the original site of the tumor and at the neck by routine check-ups, including MRI or CT, were classified as the radiosensitive group. To reduce confounding variables of the radiosensitive and radioresistant groups, we ensured that the patients in the two groups were well matched in terms of the factors closely correlated with radiosensitivity, that is, T stage, TNM, N stage, histological grade, gender, average age, radiation dose to the nasopharynx and the neck, and pretreatment hemoglobin between the two groups were not different statistically (Table 3). Tumors were staged according to the UICC 2002 cancer staging classification.

p-Ser153 RKIP was examined by immunohistochemical staining method in 90 radiosensitive and 90 radioresistant NPC tissues from enrolled patients before radiotherapy. The relationship between the rate and extent of p-Ser153 RKIP expression and the effect of radiotherapy was analyzed.

### Patient treatment

All enrolled patients received radical irradiation alone with intensity-modulated radiotherapy. Nasopharyngeal gross tumor volume (GTVnx) and the gross tumor volume of positive neck lymph nodes (GTVnd) included all gross diseases visualized on imaging examinations, such as CT and/or MRI. CTV-1 was defined as the high-risk clinical target volume that includes GTVnx plus a margin of 5 mm to 10 mm and the entire nasopharyngeal mucosa plus 5 mm submucosal volume. CTV-2 was designed for potentially involved regions. The regions not only included the posterior part of nasal cavity, posterior wall of sinus maxillaris, anterior third of clivus and cervical vertebra, and skull base; the region also included parapharyngeal space, pterygopalatine fossa, posterior ethmoid sinus, inferior sphenoid sinus, and cavernous sinus, as well. CTV-2 also included the retropharyngeal lymph nodal regions from the cranial base to lower edge of the second cervical vertebra. The CTV of the neck nodal regions (CTV-nd) included levels II, III, IV, and V, which were contoured according to the recommended principle by RTOG CTV delineation protocol for head and neck malignancies [19].

The planning target volume (PTV) was obtained based on each volume with an additional 3 mm margin and considering setup variability. Critical normal structures, which include not only the spinal cord, brainstem, optic nerves, optic decussation, lens, and eyeballs but also lobi temporalis, pituitary gland, temporomandibular joints, mandibular bone, and parotid glands, were contoured and set as organs at risk (OARs) during optimization of a treatment plan. The radiation doses prescribed in the protocol was as follows: a total dose of 68 Gy in 30 fractions at 2.27 Gy per fraction to the PTV of GTVnx, 60 Gy to 66 Gy to the PTV of the GTVnd for positive cervical lymph nodes in 30 fractions, 60 Gy at 2 Gy per fraction to the PTV of CTV-1, and 54 Gy at 1.8 Gy per fraction to the PTV of CTV-2. All patients were treated with one fraction daily for 5 days each week. The dose received by each OAR should be less than its maximum tolerance limit according to the RTOG 0225 protocol.

### Tissue microarray construction

A tissue microarray (TMA) was constructed with archival formalin-fixed, paraffin embedded tissues from 733 primary NPC and 49 normal nasopharyngeal epithelial tissue (NNET) samples. NNET samples were obtained from patients with biopsy-proven inflammatory diseases. First, after a pathologist reviewed the slides stained with hematoxylin and eosin and the localized tumor/normal areas, a tissue core from the donor block was punched by using a hollow needle with an inner diameter of 1.5 mm. Then, samples from each tissue were precisely arrayed in a recipient block at specifically assigned locations. TMA blocks were sliced into 4 μm sections embedded in slides for immunohistochemical (IHC) staining.

### Immunohistochemical staining of tissue microarray

The p-Ser153 RKIP expression levels were checked by using a semi-quantitative IHC assay. TMA staining was performed with an avidin biotin procedure following manufacturer's kit instructions by using standard two-step indirect immunohistochemistry, which is similar to that in previously described experiments [16]. The primary antibody used was a rabbit anti-human p-Ser153 RKIP monoclonal IgG (clone number, EP2845Y, rabbit anti-human, ab75971, USA) at 1:250 dilution. We used positive controls, which consisted of lung cancer samples that were previously exhibited to be stained with this antibody [15]. Tris-buffered saline in place of the primary antibody was used as a negative control.

### Evaluation of immunohistochemical staining

Similarly in previously described experiments [12], staining intensity for each section was scored by using the following scale: no staining as 0 points, mild staining as 1 point, moderate staining as 2 points, and intense staining as 3 points. A positively stained proportion was scored using the following scale: 1 point for 1 % to 24 %, 2 points for 25 % to 49 %, 3 points for 50 % to 74 %, and 4 points for 75 % to 100 %. The intensity and proportion of the ten fields-of-view were first averaged, respectively, prior to scoring. The final score was obtained by multiplying the two scores, thus forming a scale of 0, 1, 2, 3, 4, 6, 8, 9, and 12 points. 0 ~ 1 point was defined as (−), 2 ~ 4 points as (+), 6 ~ 8 points as (++), and 9 ~ 12 points as (+++). Two independent histopathologists were assigned to conduct the scoring. The histopathologists were blinded to the clinicopathological characteristics, including the received treatment and treatment outcome, of all patients.

### Statistical analysis

Our observations ended on December 31, 2012. The primary endpoint of the current study was LRRFS. The secondary endpoints were progression-free survival (PFS) and overall survival (OS). The duration of time to locoregional relapse was measured from the date of the start of radiotherapy until documented treatment failure. PFS was assessed from the start of treatment to the first defined event of failure, i.e., locoregional relapse and/or distant metastasis in patients who completely responded to radiation therapy, as well as the definite progression of disease in patients who partially responded. The duration of overall survival was calculated from the start of radiation therapy until death from any cause or until the date of the last follow-up visit for patients still alive. These endpoints were analyzed and compared using the Kaplan-Meier method and log-rank test. Multivariate analyses with the Cox proportional hazards regression model were used to determine the prognostic value of p-Ser153 RKIP expression.

The comparison of each matched factor between patients in the two groups was assessed by Pearson’s chi-square test or Students’ test. The differences in the two groups in terms of positive p-Ser153 RKIP expression of rate and extent were assessed by Pearson’s chi-square test or Fisher’s exact test. The correlation between p-Ser153 RKIP protein expression and the response to radiotherapy was analyzed by Spearman’s rank correlation test. The statistical 2-sided test was conducted by SPSS 16.0 software.

## Results

### Follow-up

The median follow-up duration was 63 months (range, 4 months to 84 months) for all patients and 84 months (range, 10 months to 84 months) for the surviving patients. The one-year, three-year, and five-year follow-up rates were 97.2 %, 96.3 %, and 92.7 %, respectively. During the follow-up period, a total of 155 patients developed locoregional recurrence, and the locoregional recurrence rate was 21.11 % (155/733). Original data can be acquired in the Additional file 1.

### p-Ser153 RKIP expression in NNET and primary NPC tissues

**Fig. 1:**
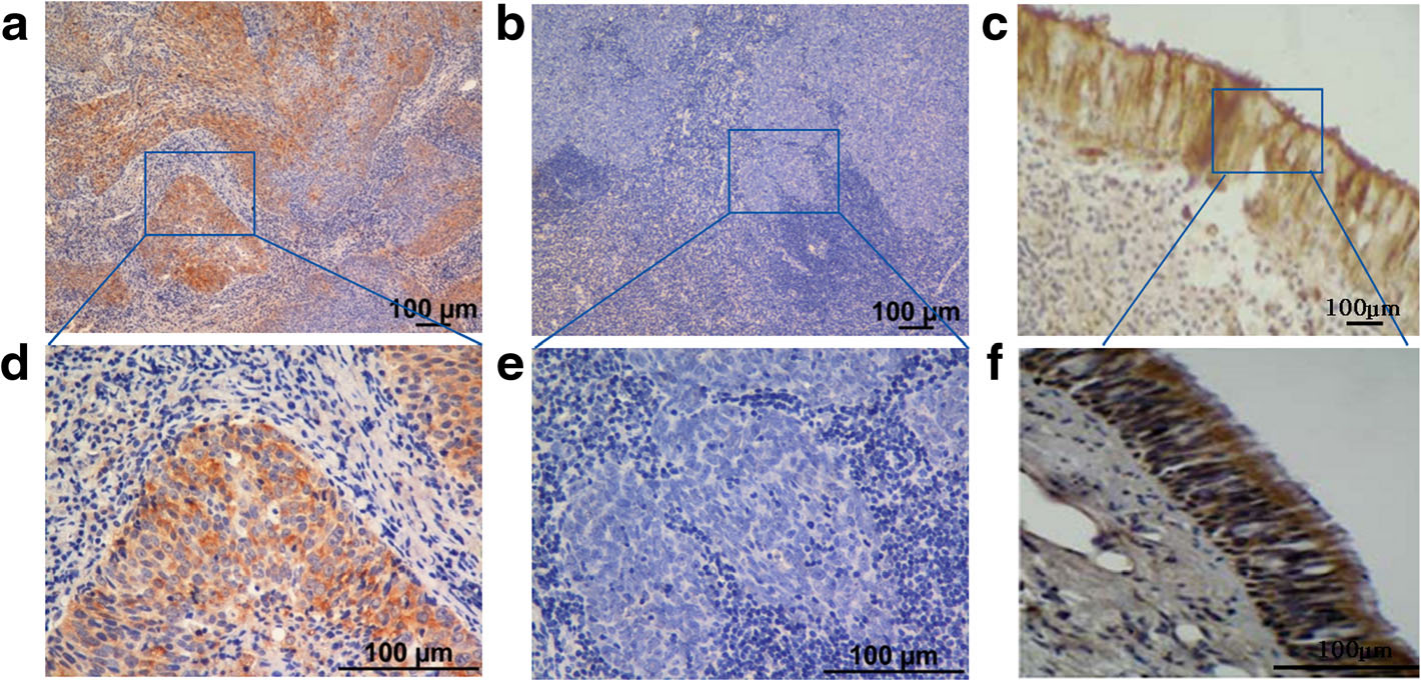
Immunohistochemical staining of p-Ser153 RKIP expression in NNET and primary NPC tissues. **a**, **d** Strongly and positively stained representative in primary NPC tumors in the radiosensitive group (100×). **b**, **e** Weakly and positively stained representative in primary NPC tumors in the radioresistant group (100×). **c**, **f** Strongly and positively stained representative in NNET (100×)

The IHC staining results revealed that strongly positive p-Ser153 RKIP staining was observed in the cytoplasm of NPC tumor cells for 80.0 % (72/90) of patients in the radiosensitive group (Fig. 1a and d) and NNET (Fig. 1c and f); by contrast, negative p-Ser153 RKIP staining was observed for 73.3 % (72/90) of patients in the radioresistant group (Fig. 1b and e). Of the 733 enrolled patients, 391 (53.3 %) showed positive p-Ser153 RKIP expression. The correlation of p-Ser153 RKIP expression with clinical characteristical factors was shown in Table 1.

**Table 1:** The correlation of p-Ser153 RKIP expression with clinical characteristical factors

Characteristic	Total		p-Ser153 RKIP expression				
	(*n* = 733)		Negative		Positive		*P* value
	No.	%	No.	%	No.	%	
Age, y(median)							
≤46	362	49.39	164	45.3	198	54.7	0.468
>46	371	50.61	178	48.0	193	52.0	
Sex							
Male	394	53.75	168	42.6	226	57.4	0.019
Female	339	46.25	174	51.3	165	487	
N classification							
N_0+1_	377	51.43	174	46.2	203	53.8	0.778
N_2+3_	356	48.57	168	47.2	188	52.8	
T classification							
T_1+2_	438	59.75	188	42.9	250	57.1	0.014
T_3+4_	295	40.25	154	52.2	141	47.8	
TNM classification							
I + II	335	57.57	160	47.8	175	52.2	0.583
III + IVa + b	398	42.43	182	45.7	216	54.3	
Pathologic classification							
Type I	10	1.36	4	40	6	60	0.116
Type II	84	11.32	48	57.1	36	42.9	
Type III	639	87.31	290	45.4	349	54.6	
Survival status							
Yes	415	56.62	130	31.3	285	68.7	<0.001
No	318	43.38	212	66.7	106	33.3	
Local-regional relapse							
Yes	155	21.15	92	59.4	63	40.6	0.001
No	578	78.85	250	43.3	328	56.7	
Post-treatment progression							
Yes	324	44.20	197	60.8	127	39.2	<0.001
No	409	56.80	145	35.5	264	64.5	
Post-treatment distant metastasis							
Yes	224	30.56	125	55.8	99	44.2	0.001
No	509	69.44	217	42.6	292	57.4

### Effect of p-Ser153 RKIP expression on survival for all of the enrolled patients

**Fig. 2:**
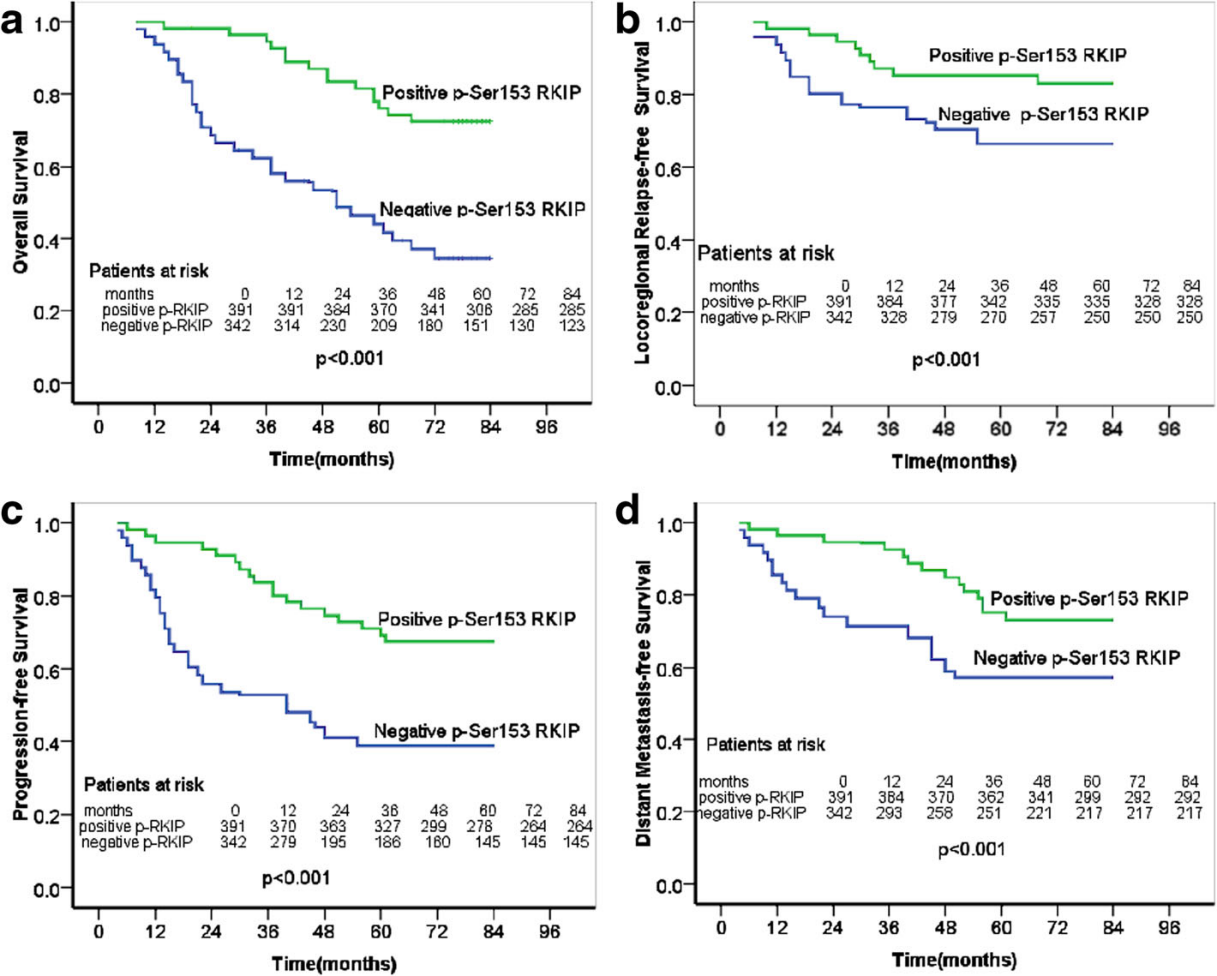
Kaplan–Meier survival curve of NPC patients on the basis of the p-Ser153 RKIP expression levels of **a** OS; **b** LRRFS; **c** PFS; and **d** DMFS

The five-year OS rates were 76.1 % and 44.1 % for the p-Ser153 RKIP positive-expression group and the p-Ser153 RKIP negative-expression group, respectively. The difference between the two groups in terms of five-year OS was significant on the basis of log-rank test (*p* < 0.001, Fig. 2a). We also analyzed the five-year LRRFS, DMFS, and PFS of the patients in the p-Ser153 RKIP positive-expression group and the p-Ser153 RKIP negative-expression group. The five-year LRRFS were 85.3 % and 66.5 % (*p* < 0.01, Fig. 2b) in the two groups, respectively. Likewise, a significant difference between the two groups was observed in terms of five-year DMFS (75.1 % versus 57.2 %, *p* = 0.023, Fig. 2c). Similarly, the five-year PFS were 69.3 % and 38.8 % (*p* < 0.01, Fig. 2d) in the two groups, respectively

In addition to irradiation dose to the neck or the primary nasopharyngeal site, sex, age, TNM stage, T stage, N stage, and p-Ser153 RKIP expression were further introduced to the Cox regression model by using the Enter method. The results revealed that positive p-Ser153 RKIP expression was an independent prognostic factor of LRRFS (HR = 0.133, CI = 0.034-0.530, *p* = 0.004) and OS (HR = 0.226, CI = 0.118-0.432, *p* <0.01) in Table 2. And it was also an independent prognostic factor of PFS (HR = 0.249, CI = 0.125-0.493, *p* <0.01) and DMFS (HR = 0.093, CI = 0.025-0.346, *p* <0.01).

**Table 2:** Matched pair comparison of clinical characteristics between the radiosensitive group and the radioresistant group. Multivariate analysis of Cox proportional hazards model for 5-year LRRFS, OS, PFS, and DMFS (*n* = 733)

Endpoints	Prognostic factors	HR	95.0 % CI	*p* value
LRRFS	p-Ser153 RKIP expression(positive versus negative)	0.133	0.034-0.530	0.004
	TNM classification(I + II versus III + IV_A+B_)	0.310	0.092-1.047	0.059
	N classification (N_0+1_ versus N_2+3_)	0.276	0.112-0.677	0.005
	T classification (T_3+4_ versus T_1+2_)	3.594	1.199-10.779	0.022
	Sex (female versus male)	0.144	0.023-0.878	0.056
	Age (<46 year versus ≥46 year)	1.075	0.992-1.164	0.077
	Absolute irradiation dose to neck (Gy)	0.347	0.097-0.910	0.066
	Absolute irradiation dose to primary nasopharyngeal site(Gy)	0.211	0.079-0.563	0.062
OS	p-Ser153 RKIP expression(positive versus negative)	0.226	0.118-0.432	<0.01
	N classification(N_0+1_ versus N_2+3_)	0.196	0.055-0.441	0.085
	T classification(T_3+4_ versus T_1+2_)	2.525	1.083-5.888	0.032
	Sex (female versus male)	1.132	1.003-1.362	0.191
	Age(<46 versus ≥ 46)	1.032	1.003-1.062	0.271
	TNM classification (I + II versus III + IV_A+B_)	0.310	0.092-1.047	0.059
DMFS	p-Ser153 RKIP expression(positive versus negative)	0.093	0.025-0.346	<0.01
	N classification(N_0+1_ versus N_2+3_)	0.122	0.048-0.425	0.010
	T classification (T_3+4_ versus T_1+2_)	8.656	1.037-72.232	0.046
	Sex (female versus male)	1.032	1.002-1.326	0.121
	Age(<46 versus ≥ 46)	1.012	1.013-1.072	0.371
	TNM classification (I + II versus III + IV_A+B_)	0.310	0.092-1.047	0.049
PFS	p-Ser153 RKIP expression(positive versus negative)	0.249	0.125-0.493	<0.01
	N classification(N_0+1_ versus N_2+3_)	0.226	0.118-0.432	<0.01
	Sex (female versus male)	2.886	0.987-8.439	0.053
	T classification (T_3+4_ versus T_1+2_)	3.525	1.083-6.27	0.032
	Age(<46 versus ≥ 46)	1.032	1.003-1.262	0.318
	Absolute irradiation dose to neck (Gy)	0.747	0.497-1.100	0.066
	Absolute irradiation dose to primary nasopharyngeal site(Gy)	0.411	0.079-0.763	0.062

*Abbreviations*: *p-Ser153 RKIP* RKIP in phosphorylated form at residue serine153, *RKIP* Raf kinase inhibitory protein, *HR* hazard ratio, *CI* confidence interval, *PFS* progression-free survival, *OS* overall survival, *DMFS* distant metastasis-free survival, *LRRFS* locoregional relapse-free survival

### The matched pair comparison of clinical characteristics between the radiosensitive group and radioresistant groups

As is shown in Table 3, most of the well-known factors that influence NPC radiosensitivity between the radiosensitive group and the radioresistant group were well-balanced in the current study. These factors included clinical staging, radiation dose, anemia, tumor differentiation degree, and certain other factors.

**Table 3:** Multivariate analysis of the five-year Cox proportional hazard model of LRRFS; OS; DMFS; and PFS. The matched pair comparison of clinical characteristics between the radiosensitive group (*n* = 90) and radioresistant group (*n* = 90)

Items		Radiosensitive group	Radioresistant group	*P*
T Stage	T1(cases)	14	11	>0.05
	T2(cases)	36	33	
	T3(cases)	28	32	
	T4(cases)	12	14	
N Stage	N0(cases)	26	23	>0.05
	N1(cases)	36	39	
	N2(cases)	23	20	
	N3(cases)	5	8	
TNM Stage	I(cases)	14	10	>0.05
	II(cases)	35	34	
	III(cases)	29	32	
	IV(cases)	12	14	
WHO Classification	I (cases)	12	14	>0.05
	II(cases)	24	28	
	III(cases)	54	48	
Sex	Man(cases)	49	55	>0.05
	Female(cases)	41	35	
Age	($\overline{x}\pm s$)(cases)	44.5 ± 14	49.0 ± 12	>0.05
Preatment Hemoglobin(g/L) ($\overline{x}\pm s$)		138.5 ± 13.2	135.8 ± 15.5	>0.05
Total irradiation dose of primary nasopharyngeal carcinoma (Gy) ($\overline{x}\pm s$)		72.5 ± 4.2	72.6 ± 3.8	>0.05
Total irradiation dose of positive neck lymph node (Gy) ($\overline{x}\pm s$)		62.2 ± 2.3	64.5 ± 4.2	>0.05
Preventive irradiation dose of negative neck lymph node (Gy) ($\overline{x}\pm s$)		54.2 ± 3.3	53.5 ± 4.3	>0.05

### Relationship between p-Ser153 RKIP expression and radiation response

The positive rate of p-Ser153 RKIP expression in the radiosensitive group versus the radioresistant group was 80.0 % versus 26.7 %. The rates of the positive p-Ser153 RKIP expression in primary NPC significantly differed between the groups (*χ*^*2*^ = 51.429, *p* < 0.01; Table 4). The staining scores of the p-Ser153 RKIP expression were significantly different between the two groups (*χ*^*2*^ = 12.498, *p* < 0.01, Table 4). Spearman analysis revealed that the intensity and rate of the the p-Ser153 RKIP protein expression was negatively correlated with the radioresistance of NPC (*r* = −0.344, *r* = −0.535, respectively, *p* < 0.01 for both groups).

**Table 4:** The Expression of the p-Ser153 RKIP protein in the radiosensitive group and the radioresistant group

p-Ser153 RKIP	The radioresistant group (*n* = 90)	The radiosensitive group (*n* = 90)	No. of patients	*p*
-	66	18	84	P* < 0.01
+	11	10	21	P** < 0.01
++	8	24	32	
+++	5	38	43	

*Abbreviations*: *RKIP* Raf kinase inhibitory protein, *p-Ser153 RKIP* RKIP in phosphorylated form at residue serine153, *Ser* serine

Note: P*, the p-Ser153 RKIP-positive expression versus the p-Ser153 RKIP–negative expression; P**, the comparison among 3 different staining scores of the p-Ser153 RKIP-positive expression

### p-Ser153 RKIP discrimination of NPC response to radiation

Based on the expression of p-Ser153 RKIP as a marker predicting radio-resistance, sensitivity, specificity, accuracy, positive predictive value, and negative predictive value were 80 % (72/90), 73.3 (66/90), 76.7 % [(72 + 66)/180], 75 % (72/96), and 78.6 % (66/84), respectively. The false positive and false negative rates were predicted as 26.7 % (24/90) and 20.0 % (18/90), respectively.

## Discussion

Limited data are available to investigate the clinical implication of the phosphorylated RKIP in tumors [15–18]. However, the relationship of p-Ser153 RKIP expression with the effect of radiation and prognosis of NPC patients has yet to be described. Thus, we should explore the clinical implication of the phosphorylated RKIP in NPC. In this study, we found for the first time that positive p-Ser153 RKIP was a favorable prognostic factor for patients who received radiation alone and that the endemic NPC patients with positive p-Ser153 RKIP expression in a NPC tissue microarray before treatment benefited from irradiation alone in terms of LRRFS. This validation was based on the patients from an established retrospective cohort composed of 733 cases. In the ongoing matched pair study and through detection by using simple IHC staining, p-Ser153 RKIP was positively correlated with radiosensitivity to NPC. Moreover, we found that p-Ser153 RKIP could be used as a biomolecular marker with good availability and authenticity to preliminarily screen the clinical radiosensitivity of NPC.

NPC is one of the most common malignancies in South China. In general, NPC is highly radiosensitive, and radical radiation is one of the most effective treatment methods against NPC. IMRT is a major breakthrough in the treatment of NPC. IMRT can be applied to improve tumor control. Encouraging results of NPC treated with IMRT have been reported. However, NPC patients with T3 or T4 stage disease showed a five-year relapse rate of 12.3 %–28.2 %, even with advanced IMRT [20, 21]. Similarly, several patients with early stages of disease develop early recurrence even with a sufficient radiation dose. Local control probability can vary among patients with NPC with the same staging, radiation dose, and differentiation grade of tumor. This finding indicates the presence of a radiosensitivity determinant. On the other hand, how to find the radiosensitivity predictors by precise, rapid, and economical methods is also a challenge. Some methods can be used to detect intrinsic radiosensitivity. However, they were complex, time-wasting. Precise, rapid, and economical methods used to predict intrinsic radiosensitivity are insufficient. Therefore, the exploration of numerous novel methods are currently underway.

Li and his colleagues [22] found that tumor recurrence time after radiotherapy can be used to evaluate radiosensitivity, which possessed a certain value in clinical practice. However, this method according to recurrence time alone after finishing radiation can only indirectly indicate radiosensitivity. Moreover, this method could be interfered by several clinopathological factors influencing radiation outcome, such as clinical staging, radiation dose, anemia, differentiation grade of tumor and so on. Therefore, in the settings, radiosensitivity cannot be precisely predicted.

As is known to us, intrinsic radiosensitivity determinant in NPC is a functional gene and/or its expressing protein, which bear certain connection with the proliferation and/or apoptosis of cancer cells. Several studies on intrinsic radiosensitivity at the molecular level can accurately reflect radiosensitivity against cancer. Therefore, an increasing number of oncologists have attached importance to these radiosensitivity determinants.

RKIP, a well-established metastasis suppressor, has been identified as a negative regulator of survival signals. RKIP is shown to be a prognostic marker in the pathogenesis of several non-head–neck cancers [7–11]. In NPC, Ruan and our previous studies had revealed that RKIP protein was not only associated with the progression and prognosis of NPC but also to NPC radiosensitivity [3, 12]. Patients with radioresistant NPC presented different RKIP expression levels from those with radiosensitive tumor. The overexpression of RKIP reverses tumor cell resistance to apoptosis by various factors, such as irradiation [2, 3], chemotherapeutic drugs [4, 5], and TRAIL [6].

However, the effect of p-RKIP expression on prognosis of NPC has yet to be elucidated. The inhibitory activity of RKIP on the Raf-1/MEK/ERK pathway is partially regulated by PKC-induced phosphorylation of RKIP at serine 153 [13]. Therefore, we hypothesized that the phosphorylated form of RKIP at the S153 residue (p-Ser153 RKIP) may be related to NPC prognosis. Previous reports showed that p-Ser153 RKIP dissociates RKIP from Raf-1, reversing the inhibitory function [13, 14]. Moreover, p-RKIP binds to G protein–coupled receptor kinase-2 (GRK-2) and dissociates it from G protein–coupled receptors (GPCRs), thus inhibiting GRK-2-mediated phosphorylation of GPCRs. This inhibition further prevents both receptor internalization and inactivation, as well as promotes cell growth and survival [14]. These results provide several theoretically convincing evidences to support the concept that p-Ser153 RKIP may be recognized as a positive regulator of survival signals, in contrast to RKIP. Interestingly, our present data do not agree with these findings. In other words, positive p-Ser153 RKIP expression is a strong predictor of favorable outcome. Relatively higher levels of p-Ser153 RKIP could predict better survival in comparison with relatively lower expression. Similarly, our findings are in agreement with the functional activities of p-Ser153 RKIP in breast cancer and lung cancer [15, 16]. In several other tumors, such as MM and stage II colon cancer, p-Ser153 RKIP may contribute positively to overall cell survival and drug resistance [17, 18]. The prognostic differences of p-Ser153 RKIP in different tumors is worth further study.

Previous studies have shown that a high or positive RKIP expression is related to the favorable prognosis in endemic-NPC [7–12]. We demonstrated that a positive p-Ser153 RKIP expression is also related to the favorable prognosis in the current study. As a possible antagonist of RKIP [13, 14], why does p-Ser153 RKIP expression also result in the beneficial outcomes, which is similar to RKIP? There is no definite explain for the findings. Firstly, Sam Cross-Knorr [18] reported that nuclear p-Ser153 RKIP-high expression is associated with poor prognosis whereas cytoplasmic p-Ser153 RKIP-high expression is associated with better prognosis in stage II colon cancer patients, which indicates that p-Ser153 RKIP might play a quite different role from cytoplasm to nuclei. So we speculate that good prognostic implications of p-Ser153 RKIP in our current study may be associated with its expression in cytoplasm but not in nuclei in NPC. Since the majority of studies[7–11] including our previous studies[12] showed the staining of RKIP positive NPC samples was in cytoplasm but not in nuclei, we furtherly speculate a dominant proportion of RKIP expression may be in the p-Ser153 RKIP form. Secondly, Al-Mulla F et al. [16] also found that both RKIP and p-Ser153 RKIP are related to the favorable prognosis in a study on ductal breast cancer, which is similar to our results. All these data demonstrated it is not impossible that both RKIP and p-Ser153 RKIP are related to the favorable prognosis in NPC.

The relationship between p-RKIP expression levels and radiosensitivity to NPC has yet to be reported. In the present study, we found a differential expression of p-Ser153 RKIP protein between the radiosensitive group and the radioresistant group. More importantly, we further validated that pretreatment positive p-Ser153 RKIP expression levels are positively associated with NPC radiosensitivity (*r* = 0.535, *p* < 0.01). This finding is similar to the function of RKIP in terms of NPC radiosensitivity [3].

p-RKIP expression status has yet to be reported as an indicator of preliminarily screening radiosensitivity to NPC. We found that p-Ser153 RKIP expression can logically predict radiosensitivity of NPC using p-Ser153 RKIP as a maker. Sensitivity, specificity, accuracy, positive predictive value, and negative predictive value were predicted as 80.0 %, 73.3 %, 76.7 %, 75.0 %, and 87.6 %, respectively, all amounting to 73.0 %. Liu et al. [23] found that COX-2 expression is a good predictor of NPC radiosensitivity, with good sensitivity of 86.67 %, specificity of 63.3 %, accuracy of 75.0 %, positive predictive value of 70.27 %, and negative predictive value of 82.6 %. Contrastingly, in the current study, specificity of 73.3 %, accuracy of 76.7 %, and positive predictive value are superior to those in previous results, but sensitivity and negative predictive value are poorer. The used method, IHC staining on a NPC tissue microarray, is more simple and practical. We conclude that p-Ser153 RKIP-positive expression could serve as a biomarker in the preliminarily screening of the intrinsic radiosensitivity of NPC.

The retrospective study matched the same period. In the study, most main factors influencing radiosensitivity, i.e., clinical staging, radiation dose, anemia, tumor differentiation degree, and certain other factors between two matched groups were well-balanced to the maximum extent. Our objective is to balance the factors that interfere with intrinsic radiosensitivity between two groups. Thus, a difference between two matched groups should be consistent with intrinsic radiosensitivity, as anticipated. Therefore, our study results must be authentic.

Most of patients with negative p-Ser153 RKIP expression are radio-resistant in the current study. These patients showed inferior effectiveness when radiation alone is given, presenting a relapse rate of about 30.0 % within five years. If a more aggressive treatment is combined with chemotherapy, molecular targeted therapy, and immunotherapy from the beginning of treatment, five-year survival may be improved.

In the previous study, RKIP can inhibit the radiation resistance of cancer caused by Raf-1[24]. RKIP activity was found to be under the control of post-translational modification, which involves PKC-mediated phosphorylation at serine 153 [13]. In the current study, pretreatment positive p-Ser153 RKIP expression levels showed negative correlation with radio-resistance to NPC and could serve as a biomarker in the preliminary screening of the intrinsic radiosensitivity of NPC. Therefore, we speculate that any available means that regulate the post-translational modification of RKIP at serine 153 may be a target for therapeutic intervention. For example, p-Ser153 RKIP-inductive agents and/or PKC-regulators may partially enhance tumor radiosensitivity and improve clinical outcome.

Our concurrent research found that p-Ser153 RKIP protein expression can act as an effective maker for use in radiosensitivity prediction with higher sensitivity. However, the false positive rate of 26.7 % is still high as a predictor. This single marker in predicting radiosensitivity remains to be improved. Therefore, we should explore more predictors of radiosensitivity. Moreover, we confirm that sensitivity and/or specificity could be improved when p-Ser153 RKIP is combined with other indicators.

## Conclusions

In conclusion, using a NPC tissue microarray with immunohistochemistry staining, we found for the first time that positive p-Ser153 RKIP was favorable prognostic factor for patients who received radiation alone and also positively correlated with NPC radiosensitivity. The expression could be used as a biomolecular marker with good availability and authenticity to preliminarily screen the internal radiosensitivity of NPC.

## Additional files

Additional file 1: Original data from 733 of the enrolled patients. (RAR 11 kb) Additional file 2: All sources of funding for the research reported. (RAR 31261 kb)

## Abbreviations

CT: Computed tomography
DMFS: Distant metastasis-free survival
IMRT: Intensity-modulated radiotherapy
LRRFS: Locoregional relapse-free survival
MRI: Magnetic resonance imaging
NPC: Nasopharyngeal carcinoma
OS: Overall survival
PFS: Progression-free survival
p-Ser153 RKIP: RKIP in a phosphorylated form at residue serine153
RKIP: Raf kinase inhibitor protein
RT: Radiotherapy
RTOG: Radiation therapy oncology group
UICC: International Union Against Cancer
WHO: World Health Organization
